# Active and Fit Communities. Associations between Neighborhood Walkability and Health-Related Fitness in Adults

**DOI:** 10.3390/ijerph17041131

**Published:** 2020-02-11

**Authors:** Gavin R. McCormack, Levi Frehlich, Anita Blackstaffe, Tanvir C. Turin, Patricia K. Doyle-Baker

**Affiliations:** 1 Department of Community Health Sciences, Cumming School of Medicine, University of Calgary, Calgary, AB T2N 1N4, Canada; lcfrehli@ucalgary.ca (L.F.); ambortni@ucalgary.ca (A.B.); 2 Department of Family Medicine, Cumming School of Medicine, University of Calgary, Calgary, AB T2N 1N4, Canada; chowdhut@ucalgary.ca; 3 Faculty of Kinesiology, University of Calgary, Calgary, AB T2N 1N4, Canada; pdoyleba@ucalgary.ca

**Keywords:** fitness, exercise, muscular strength, flexibility, cardiorespiratory, walkability, parks, neighborhood

## Abstract

There are many health benefits of regular physical activity and improving physical fitness levels can reduce the risk of chronic disease. Accumulating evidence suggests the neighborhood built environment is important for supporting physical activity; however, few studies have investigated the contribution of the neighborhood built environment to fitness levels. We examined the associations between objectively-determined and self-reported neighborhood walkability and overall and specific components of perceived health-related fitness (cardiorespiratory, muscular strength, and flexibility) in a random sample of 592 adults from two areas of Calgary (Canada). Participants provided complete data to an online questionnaire capturing perceived cardiorespiratory fitness (CRF), muscular strength (MST), flexibility, moderate-to-vigorous intensity physical activity (MVPA), resistance training, and sociodemographic characteristics. The questionnaire also captured participant’s perceptions of their neighborhood’s walkability (Physical Activity Neighborhood Environment Scale; PANES) and the physical activity supportiveness of neighborhood parks (Park Perceptions Index; PPI). Objectively-measured neighborhood walkability was estimated using Walk Score^®^. The average (SD) age of participants was 46.6 (14.8) years and 67.2% were female. Participants, on average, participated in at least 30-minutes of MVPA on 3.4 (2.1) days/week and undertook resistance training 2.0 (1.8) days/week. Adjusting for covariates, Walk Score^®^ was not associated with any fitness outcomes. Adjusting for covariates, the PANES index was positively associated (*p* < 0.05) with CRF, MST, flexibility, and overall fitness and the PPI was positively associated (*p* < 0.05) with all fitness outcomes except MST. Our findings provide novel preliminary evidence suggesting the neighborhood built environment may be important for supporting higher health-related fitness levels in adults.

## 1. Introduction

Improving physical fitness levels can prevent chronic disease and promote health and wellbeing [1,2]. Physical fitness is multidimensional, reflecting an individual’s cardiorespiratory or aerobic capacity (CRF), muscular strength (MST), flexibility, agility, power, and speed [1,2]. CRF reflects an individual’s ability to undertake continuous whole-body, submaximal, physical activity over an extended duration [1,3]. Globally, levels of CRF have steadily declined during the last 50 years [4]—of concern given that CRF protects health independent of physical activity levels [5,6]. Notably, higher CRF is associated with a reduced risk of metabolic syndrome [5,6,7], type 2 diabetes [5,8], adverse cardiovascular-related outcomes [9,10,11,12], and depression [13].

MST reflects an individual’s ability to exert maximal or near-maximal force of a muscle group against an external resistance [1,3]. Higher MST is associated with a reduced risk of all-cause mortality [14,15,16], weight gain [17], adverse cardiovascular-related outcomes [11,15,18], metabolic syndrome [7,19], injury [20], and better self-reported health [21] and quality of life [22]. Development and maintenance of MST is particularly important among older adults to reduce the risk of chronic disease (e.g., osteoporosis), falls, physical impairment, and to counteract sarcopenia [23]. Notably, independent of CRF and physical activity, MST protects against all-cause and cardiovascular-related mortality [15]. Furthermore, higher levels of both upper (e.g., handgrip) and lower body (e.g., knee extension) strength are associated with better health [16]. Flexibility relates to mobility and the functional capacity of joints to move through a full range of motion [1,3]. Despite weak support for the health and functional benefits of flexibility [24], some findings suggest higher flexibility might be associated with reduced injury risk, improved ability to undertake tasks of daily living and independence, mobility, and improved quality of life [22,25].

Evidence demonstrating associations between the neighborhood environment and chronic disease [26,27], well-being [28], and physical activity [29,30] exists, yet few studies have investigated the relations between the neighborhood built environment and fitness [31,32,33,34,35]. For example, Hoehner et al. [31] found positive associations between objectively-measured neighborhood intersection density, vegetation coverage, and count of private exercise facilities and objectively-measured CRF. In another study, Hoehner et al. [35] found that neighborhoods with older homes and shorter commute times were positively associated with objectively-measured CRF. Shaffer et al. [33] found no significant correlations between perceived neighborhood built characteristics and objectively-measured CRF among college students, however, seeing others exercising in the neighborhood was positively correlated with push-up performance, seeing others active in the same apartment complex was positively correlated with curl-up performance, and the count of resources in the apartment was negatively correlated with curl-up performance. To our knowledge no studies have investigated the associations between overall neighborhood walkability and fitness levels.

Performance on exercise tolerance testing with either treadmill or cycle ergometers in laboratory settings or field-based tests (e.g., shuttle run, walking tests, step tests) are typically used to estimate CRF [3,36]. Static and dynamic MST can be measured using tests that elicit isometric, concentric, eccentric, or isokinetic contractions involving various laboratory and field-based protocols (e.g., bodyweight exercises, resistance machines, free weights, dynamometers, force plates) [3,16,36,37,38]. Flexibility is often assessed using goniometry [25], observer visual rating of range of motion [39], sit-and-reach distance [25], or other tests of functional mobility [3,39]. However, in epidemiological studies, objective measurement of fitness levels may not always be feasible due to the recruitment of large geographically dispersed samples, and the limited availability of expertise and specialized equipment to undertake these physiological fitness tests. In these large samples, measuring self-reported or “perceived” fitness is a useful alternative. Despite methodological issues of perceived fitness measures (i.e., overestimation), [40,41] moderate-to-strong positive associations between perceived and objectively-measured fitness have been found [41,42]. Similar to objectively-measured fitness levels, perceived fitness is associated with better weight status [42,43,44,45], lower total and higher HDL cholesterol [43,45], lower resting heart [43,45], lower cardiorespiratory disease risk [42], and lowered risk of all-cause mortality [46]. Positive associations between leisure-time physical activity and perceived fitness have also been found [44,47,48].

The aim of this study was two-fold: (1) To examine the associations between objectively-determined and self-reported neighborhood walkability and overall and specific components of perceived health-related fitness (CRF, MST, and flexibility), and; (2) to examine the extent to which moderate-to-vigorous physical activity (MVPA) and resistance training attenuate the associations between objectively-determined and self-reported neighborhood walkability and perceived health-related fitness. Given that physical activity is associated with fitness levels, and the built environment is associated with physical activity, we hypothesize that physical activity should explain some of the association estimated between the built environment variables and fitness outcomes.

## 2. Materials and Methods

### 2.1. Study Design and Recruitment

In August and September 2018, we used a municipal generated list of residential addresses to recruit a random sample of households from two sites that consisted of established (i.e., built-out) neighborhoods in Calgary (Alberta, Canada). Site 1 consisted of two adjacent neighborhoods located in central southeast Calgary (Inglewood and Ramsey) and Site 2 consisted of nine adjacent neighborhoods located in central southwest Calgary (Glendale, Killarney, Glengarry, Richmond, Rosscarrock, Scarboro, Sunalta West, Shaganappi, and Spruce Cliff). These neighborhoods were geographically situated near locations identified by the City of Calgary to undergo major urban infrastructure changes in 2019 as part of a municipal initiative [49] to redevelop central, important, and historic streets. Based on available Civic Census data (2016) aggregated across the study neighborhoods, on average, approximately 50% of the population was female, total household median before tax income was $108,000, and 41% had completed a university level education.

We collected baseline data from households within these neighborhoods as part of an investigation on urban form and health. Randomly sampled households (Site 1 *n* = 3476 and Site 2 *n* = 3842) received two study recruitment postcards, 28 days apart, inviting one adult per household with the last birthday (≥18 years of age; with internet access; able to read and respond in English, and; no plans of moving neighborhood before August 2019) to complete an online questionnaire (SurveyMonkey^®^). In addition to other characteristics, the comprehensive questionnaire captured participants’ perceived fitness, physical activity, resistance training, perceptions of the neighborhood environment, and sociodemographic variables. All data included in the current study were self-reported.

Among the households invited, 663 participants completed the baseline online questionnaire (response rate = 9%). Of those participants, 116 participants also completed a second online questionnaire, on average 8.2 (SD 5.1) days later, to evaluate test-retest reliability of a subset of items (*n* = 103 provided complete data for the reliability analysis). Participants completing the baseline questionnaire received a $5 gift card and an additional $5 gift card for completing the second questionnaire. The University of Calgary Conjoint Health Research Ethics Board approved this study (REB18-0855).

### 2.2. Data Collection

Perceived fitness: Participants, using visual analogue scales (scored 1 to 100), reported their perceived CRF, MST, and flexibility related to others of the same age and sex. For CRF, participants scored their “aerobic fitness or stamina (ability to exercise continuously for extended periods)” from “poor” to “excellent”; their MST from “weak” to “strong”; and flexibility (or limberness) from “poor” to “excellent”. These items have demonstrated reliability and validity [41,42,50,51,52]. In our sample, test-retest reliability (intra-class correlation (ICC) and 95 percent confidence intervals (95 CI)) for perceived CRF (ICC = 0.84; 95 CI 0.77, 0.89), MST (ICC = 0.75; 95 CI 0.65, 0.82), and flexibility (ICC = 0.72; 95 CI 0.61, 0.80) was acceptable. We summed and averaged the three items to create an overall perceived fitness index (PF), which also had acceptable test-retest reliability (ICC = 0.79; 95 CI 0.71, 0.85) and internal consistency (Cronbach’s α = 0.87).

Moderate-to-vigorous intensity physical activity (MVPA): A single item captured level of physical activity (i.e., frequency of days achieving at least 30-minutes of MVPA in the past week) [53]. The item has acceptable test-retest reliability and strong agreement with other single-item global measures of physical activity [53]. In our sample this item had acceptable test-retest reliability (ICC = 0.74; 95 CI 0.65, 0.82).

Resistance training: Participants reported frequency of days in a usual week they undertook physical activity or exercise to strengthen their muscles. Participants included in their estimate bodyweight (e.g., yoga, sit-ups, and push-ups) and non-body weight (e.g., weight machines, free weights, elastic bands) resistance activities, but excluded aerobic activities like walking, running, or cycling. This item had acceptable test-retest reliability (ICC = 0.74; 95 CI 0.65, 0.81).

Sociodemographic and health characteristics: Participants reported their biological sex, age, highest education level achieved (completed university vs. no university), gross annual household income (<$50,000, $50,000–99,999, $100,000–149,000, $150,000–199,999, and ≥$200,000), dog ownership (yes vs. no), motor vehicle available for personal use (always/sometimes vs. never/don’t drive), and ethnicity (Caucasian vs. other [Chinese, South Asian, Black, Filipino, Latin American, Southeast Asian, Arab, West Asian, Japanese, Korean, or Aboriginal]). Participants also reported their current use of tobacco (daily or occasionally vs. no current use).

Self-reported neighborhood built environment: The Physical Activity Neighborhood Environment Scale (PANES) [54] measured participant’s perception of their neighborhood’s walkability. Sixteen items (four-point response scales), including seven core and four recommended items, plus five optional items, captured built features including dwelling types, destinations, transit, sidewalks, bicycle infrastructure, recreational facilities, crime, traffic, connectivity, and aesthetics. The reliability of the PANES has been demonstrated [54,55,56,57] including for an online version administered with Canadian adults [58]. The PANES items were aggregated into an index (Cronbach’s α = 0.71), with higher scores representing higher perceived walkability.

In addition to the PANES, six-items (four-point response scales; strongly disagree to strongly agree) captured participants’ perceptions about neighborhood parks (i.e., there are many parks in my neighborhood, parks in my neighborhood are safe to visit at night, parks in my neighborhood are safe to visit during the day, parks in my neighborhood are attractive, most parks in neighborhood are too small for physical activity, most parks in my neighborhood include features that support physical activity). Item responses were aggregated (Cronbach’s α = 0.60), with higher scores on the park perceptions index (PPI) presenting more positive perceptions of neighborhood parks.

Objectively-determined neighborhood built environment: Walk Score^®^ (www.walkscore.com) was linked to residents’ six-digit residential postal code to estimate neighborhood walkability. Walk Score^®^ measures amenities using a distance decay function in which amenities located within a 5-minute walk receive the maximum amount of points while amenities located further than a 30-minute walk receive a score of zero. Walk Score^®^ also includes measures of population and intersection density, as well as road metrics [59]. In Canadian studies, Walk Score^®^ is found to be positively associated with other objective measures of walkability [60] and walking [61,62].

### 2.3. Statistical Analysis

We estimated descriptive statistics (mean, standard deviation, and frequencies) for all variables within our sample. Spearman rank correlations (r) were estimated for the built environment variables and for the fitness variables. Separate multivariable linear regression models estimated adjusted unstandardized beta (b) and 95 percent confidence intervals (95 CI) for the associations between each Walk Score^®^, PANES walkability, and PPI and perceived CRF, MST, flexibility, and PF. Model 1 was adjusted for age, sex, ethnicity, education, household income, dog ownership, access to a motor vehicle, and tobacco use. Model 2 further adjusted for MVPA and Model 3 adjusted for MVPA and resistance training, to assess whether MVPA and resistance training attenuated the estimated associations between the built environment and fitness variables. Statistical significance was set at an alpha of less than 0.05 and statistical analysis was undertaken using SPSS Statistics for Windows (IBM Corp., Version 25.0., Armonk, NY, USA).

## 3. Results

### 3.1. Sample Characteristics

Complete data were available for 592 participants. The sample had a mean (SD) age of 46.0 (14.7), and consisted of mostly females, Caucasians, those with a university education, and households with incomes of at least $100,000 (Table 1). Most participants had access to a motor vehicle, did not own a dog, and did not currently use tobacco. Participants reported on average participating in at least 30-minutes of MVPA on 3.4 (2.1) days/week and undertook resistance training 2.0 (1.8) days/week. Participants, on average reported moderate-to-high levels of perceived CRF (65.2 (23.4)), MST (64.9 (21.8)), flexibility (62.3 (22.8)) and PF (64.1 (19.4)) (Table 1).

### 3.2. Neighborhood Correlates of Perceived Cardiorespiratory Fitness

Walk Score^®^ was not significantly associated with CRF in any models (Table 2). Adjusting for sociodemographic covariates (age, sex, ethnicity, education, household income, dog ownership, access to a motor vehicle, and tobacco use) only, the PANES walkability index was positively associated with CRF (b 8.66; 95 CI 4.11, 13.21; *Model 1*) however, the association attenuated after adjusting for MVPA (b 6.77; 95 CI 2.72, 10.83; *Model 2*) and further adjusting for resistance training (b 6.47; 95 CI 2.44, 10.49; *Model 3*). Adjusting for sociodemographic covariates, the PPI was associated with CRF (b 6.60; 95 CI 3.06, 10.14; *Model 1*) although this estimate also attenuated after adjustment for MVPA (b 3.92; 95 CI 0.73, 7.10; *Model 2*) and resistance training (b 3.75; 95 CI 0.59, 9.91; *Model 3*).

Across all models, MVPA and resistance training were positively associated with CRF. Relative to all sociodemographic covariates and resistance training, MVPA accounted for most of the explainable variance in CRF (18.2% to 19.5%).

### 3.3. Neighborhood Correlates of Perceived Muscular Strength

Walk Score^®^ was not significantly associated with MST in any models (Table 2). Adjusting for sociodemographic covariates only, the PANES walkability index was positively associated with MST (b 7.17; 95 CI 2.89, 11.46; *Model 1*).This association however, attenuated after adjusting for MVPA (b 5.81; 95 CI 1.79, 9.82; *Model 2*) and further adjusting for resistance training (b 5.15; 95 CI 1.29, 9.01; *Model 3*). Adjusting for sociodemographic covariates, the PPI was associated with MST (b 4.33; 95 CI 0.99, 7.67; *Model 1*) but this estimate attenuated to non-significance after adjusting for MVPA (b 2.37; 95 CI −0.79, 5.53; *Model 2*) and resistance training (b 2.02; 95 CI −1.02, 5.05; *Model 3*).

Across all fully-adjusted models, MVPA and resistance training were positively associated with MST. Relative to all sociodemographic covariates and resistance training, MVPA accounted for most of the explainable variance in MST (11.2% to 12%). Resistance training accounted for 6.4% to 6.5% of the explainable variance in MST.

### 3.4. Neighborhood Correlates of Perceived Flexibility

Walk Score^®^ was not significantly associated with flexibility in any models (Table 2). Adjusting for sociodemographic covariates only, the PANES walkability index was positively associated with flexibility (b 8.26; 95 CI 3.69, 12.84; *Model 1*) however, this association attenuated after adjusting for MVPA (b 7.30; 95 CI 2.83, 11.77; *Model 2*) and after adjusting for resistance training (b 7.01; 95 CI 2.57, 11.47; *Model 3*). Similarly, the PPI was significantly associated with flexibility (b 6.12; 95 CI 2.56, 9.68; *Model 1*) which attenuated after adjusting for MVPA and resistance training (b 4.62, 95 CI 1.14, 8.11; *Model 3*).

Perceived flexibility was higher among those who reported more frequent resistance training and MVPA. MVPA accounted for 4.9% to 5.6% of explainable variance in flexibility, while resistance training accounted for about 1%.

### 3.5. Neighborhood Correlates of Overall Perceived Fitness

Walk Score^®^ was not significantly associated with overall fitness in any models (Table 2). Adjusting for sociodemographic covariates only, the PANES walkability index was positively associated with overall fitness (b 8.03; 95 CI 4.23, 11.84; *Model 1*). This association attenuated after adjusting for MVPA (b 6.63; 95 CI 3.02, 4.36; *Model 2*) and after adjusting for resistance training (b 6.21; 95 CI 2.79, 9.63; *Model 3*). The PPI was also associated with overall fitness (b 5.68; 95 CI 2.72, 8.65; *Model 1)* which attenuated after adjusting for MVPA and resistance training (b 3.46; 95 CI 0.78, 6.15; *Model 3*).

MVPA and resistance training were positively associated with overall fitness across all models. MVPA accounted for 14.7% to 15.9% and resistance training accounted for 3.1% to 3.3% of the explainable variance in overall fitness.

## 4. Discussion

Our finding of a relationship between the built environment and health-related fitness is novel. While Walk Score^®^ was not associated with perceived fitness, CRF, MST, and flexibility were higher among those who perceived walkability in their neighborhood to be higher. Notably, the relationships between perceived walkability and perceived fitness remained even after adjusting for sociodemographic characteristics, MVPA, and resistance training. Furthermore, perceived supportiveness of neighborhood park environments was also positively associated with CRF, MST, and flexibility. Similar to previous studies [31,33,35], our findings suggest that physical activity supportive built environments may support fitness levels. Population level interventions that can support adults accruing higher levels of fitness, such as modifying the built environment, is important given fitness levels are declining [4] and fitness levels protect against chronic disease [5,6].

Despite previous studies finding associations between objectively-measured built characteristics and fitness [31,35], Walk Score^®^ was not associated with perceived fitness in our study. Others have found neighborhood street connectivity, vegetation, and access to exercise facilities [31] as well as living in neighborhoods with older homes and short commute times [35] to be positively associated with fitness. Several reasons may explain the differences in findings between our study and previous studies. First, walkability in our study represented the overall urban design of the neighborhood (or combination characteristics) while in previous studies [31,35] associations were estimated between individual built characteristics and fitness. Specific objectively-measured built characteristics may be more supportive of fitness levels than the overall walkability of the neighborhood. Second, Walk Score^®^ includes access to destinations that may be more supportive of transportation versus recreational physical activity—the latter might be more supportive of fitness. However, CRF has been found to be higher among those who report walking and cycling to local amenities [34]. Third, the fitness outcomes in our study were self-reported while previous studies objectively-measured CRF [31,35]. Despite positive associations between perceived and objectively-measured fitness [41,42] unmeasured factors may impact the accuracy of self-reported fitness. Finally, our study recruited participants from two study sites, both of which included adjacent built-out neighborhoods. It is possible there was a lack of variability in Walk Score^®^ within the two sites making it difficult to detect associations, if they existed, with fitness. Sampling from geographically dispersed neighborhoods is needed to ensure there is sufficient variability in neighborhood urban form.

While objectively-measured walkability was not associated with fitness outcomes in our study, we did find associations for perceived neighborhood walkability and perceived physical activity supportiveness of parks. A previous study found positive associations between seeing others active in the neighborhood or in an apartment complex and local resources and muscular endurance [33]. Notably, our findings advance this previous research by demonstrating associations between perceived neighborhood and park environments and different components of fitness, including CRF, MST, and flexibility. Thus, built environment changes that result in positive perceptions of neighborhood and park environments could lead to improvements in fitness via increasing MVPA. Parks that are maintained, equipped with different amenities and facilities, clean, aesthetically appealing, and which are safe may encourage their use, which could lead to more physical activity [63] and opportunity to improve fitness. Similarly, neighborhoods that have functional (connectivity, sidewalks, etc.), aesthetic, destination, and safety-built features can support physical activity [64] and potentially improve health-related fitness.

Given the cross-sectional study design, we cannot infer causality between perceived fitness, physical activity, and the built environment. Similar to cross-sectional studies of the built environment and physical activity [29,65], residential self-selection may be a concern in cross-sectional studies of the built environment and fitness (fitter individuals choosing to reside in neighborhoods that include built characteristics that provide them with opportunities to undertake physical activity for the purpose of improving or maintaining their fitness levels). Further, inflated correlations between variables may have resulted from participants self-reporting most variables included in the analysis. Social desirability bias may also be present. Adults have been found to overestimate their perceived fitness [40,41]. Moreover, it is possible that our single-item measures of CRF, MST, and flexibility while having face validity are limited in their content validity. Comprehensive self-report measures of health-related fitness are needed. Nevertheless, like previous studies [41,42,50,51,52], the perceived fitness variables included in our study were reliable and associated with MVPA, resistance training, and the built environment. While not always feasible in large epidemiological studies, longitudinal and cross-sectional studies that examine the associations between the built environment and objectively-assessed fitness (laboratory or field tests) are needed.

Compared with available population statistics, our sample included more females, were more highly educated, and had higher household incomes. Based on the reported days of MVPA (≥30 minutes/day) and days of resistance training undertaken each week our sample was considered relatively active. The low study response rate and our purposive sampling of study sites and neighborhoods further limits the external validity of our findings. Notably, the r-squared values for the fully-adjusted models suggest that other factors might also be important for explaining perceived fitness. For instance, several factors are consistently associated with objectively-measured fitness, specifically CRF, such as male sex, age, education, ethnicity, weight status, resting heart rate, blood pressure, smoking, alcohol use, and physical activity [66], yet the determinants of perceived CRF could be different. Speculatively, actual fitness levels, objectively-measured physical activity, sedentary behavior, access to fitness facilitates and equipment, other built characteristics (e.g., pathways, cycle paths, fitness parks), perceptions of self-image, self-efficacy, genetics, weight status, and presence of an injury or mobility issues could be important correlates of perceived fitness.

## 5. Conclusions

Perceived health-related fitness is associated with perceived neighborhood walkability and perceived park supportiveness for physical activity. While participation in MVPA and resistance training partially explained these associations, perceptions of the built environment remained associated with perceived health-related fitness. Creating physical activity supportive neighborhoods could lead to improvements in health-related fitness levels. Notably, self-reported CRF, MST, and flexibility were all associated with perceived walkability and park supportiveness for physical activity, suggesting that different built features might impact different components of health-related fitness. Future research should explore other potentially important built environment correlates of health-related fitness to provide evidence to inform urban planning and neighborhood-based health promotion interventions.

## Figures and Tables

**Table 1:** Sample profile of sociodemographic, physical activity, built environment, and fitness variables (*n* = 592).

Characteristic	Category	Estimate
Age in years [mean ± SD]		46.0 ± 14.7
Sex [*n* (%)]	Male	194 (32.8)
	Female	398 (67.2)
Ethnicity [*n* (%)]	Caucasian	500 (84.5)
	Other ethnicities	92 (15.5)
Highest level of education [*n* (%)]	Less than university	128 (21.6)
	Completed university	464 (78.4)
Annual household income [*n* (%)]	Less than $50,000	84 (14.2)
	$50,000 to $99,999	140 (23.6)
	$100,00 to $149,999	113 (19.1)
	$150,000 to $199,999	86 (14.5)
	$200,000 or more	111 (18.8)
	Don’t know	58 (9.8)
Dog living in the home [*n* (%)]	At least one dog in home	170 (28.7)
	No dog in home	422 (71.3)
Motor Vehicle Access [*n* (%)]	Always/Sometimes	543 (91.7)
	Never/Don’t drive	49 (8.3)
Tobacco use at present time [*n* (%)]	Yes, daily or occasionally	53 (9.0)
	No current tobacco use	539 (91.0)
Number of days/week of ≥30+ min of MVPA [mean ± SD]		3.4 ± 2.1
Number of days/week of resistance training [mean ± SD]		2.0 ± 1.8
Walk Score^®^ (0 to 100) [mean ± SD]		62.3 ± 15.0
Physical Activity Neighborhood Environment Scale (1 to 4) [mean ± SD]		3.3 ± 0.4
Park Perceptions Index (1 to 4) [mean ± SD]		3.2 ± 0.5
Perceived cardiorespiratory fitness (0 to 100) [mean ± SD]		65.2 ± 23.4
Perceived reported muscle strength (0 to 100) [mean ± SD]		64.9 ± 21.8
Perceived flexibility (0 to 100) [mean ± SD]		62.3 ± 22.8
Overall perceived fitness (0 to 100) [mean ± SD]		64.1 ± 19.4

MVPA: Moderate-to-vigorous intensity physical activity.

**Table 2:** Linear regression unstandardized coefficients (b) and 95% confidence intervals (95 CI) for the association between perceived fitness, built environment, and physical activity (*n* = 592).

	Cardiorespiratory Fitness			Muscle Strength			Flexibility			Overall Fitness		
	Model 1	Model 2	Model 3	Model 1	Model 2	Model 3	Model 1	Model 2	Model 3	Model 1	Model 2	Model 3
	b (95 CI)	b (95 CI)	b (95 CI)	b (95 CI)	b (95 CI)	b (95 CI)	b (95 CI)	b (95 CI)	b (95 CI)	b (95 CI)	b (95 CI)	b (95 CI)
Walk Score^®^	−0.04 (−0.16, 0.08)	0.01 (−0.10, 0.12)	<0.00 (−0.11, 0.11)	−0.01 (−0.12, 0.11)	0.03 (−0.08, 0.14)	0.01 (−0.09, 0.12)	−0.03 (−0.15, 0.09)	−0.01 (−0.13, 0.11)	−0.01 (−0.13, 0.11)	−0.03 (−0.13, 0.08)	0.01 (−0.08, 0.11)	<0.00 (−0.09, 0.09)
MVPA (days/week)		5.05 * (4.26, 5.84)	4.57 * (3.74, 5.40)		3.68 * (2.91, 4.46)	2.69 * (1.89, 3.48)		2.63 * (1.76, 3.49)	2.18 * (1.26, 3.10)		3.79 * (3.11, 4.47)	3.15 * (2.44, 3.85)
RT (days/week)			1.62 * (0.65, 2.58)			3.37 * (2.45, 4.29)			1.50 * (0.43, 2.57)			2.16 * (1.34, 2.98)
*R^2^*	9.5	29.0	30.3	8.1	20.1	26.6	4.3	9.9	11.0	7.9	23.8	27.1
												
PANES	8.66 * (4.11, 13.21)	6.77 * (2.72, 10.83)	6.47 * (2.44, 10.49)	7.17 * (2.89,11.46)	5.81 * (1.79, 9.82)	5.15 * (1.29, 9.01)	8.26 * (3.69, 12.84)	7.30 * (2.83, 11.77)	7.01 * (2.57, 11.47)	8.03 * (4.23, 11.84)	6.63 * (3.02, 4.36)	6.21 * (2.79, 9.63)
MVPA (days/week)		4.95 * (4.17, 5.73)	4.50 * (3.68, 5.32)		3.59 * (2.81, 4.36)	2.62 * (1.84, 3.41)		2.53 * (1.67, 3.39)	2.12 * (1.21, 3.02)		3.69 * (3.02, 4.36)	3.08 * (2.38, 3.78)
RT (days/week)			1.5 5* (0.59, 2.50)			3.32 * (2.40, 4.23)			1.42 * (0.36, 2.47)			2.09 * (1.28, 2.91)
*R^2^*	11.5	30.3	31.5	9.8	21.1	27.5	6.3	11.5	12.5	10.5	25.6	28.7
												
PPI	6.60 * (3.06, 10.14)	3.92 * (0.73, 7.10)	3.75 * (0.59, 9.91)	4.33 * (0.99, 7.67)	2.37 (−0.79, 5.53)	2.02 (−1.02, 5.05)	6.12 * (2.56, 9.68)	4.77 * (1.27, 8.28)	4.62 * (1.14, 8.11)	5.68 * (2.72, 8.65)	3.69 * (0.95, 6.43)	3.46 * (0.78, 6.15)
MVPA (days/week)		4.92 * (4.13, 5.70)	4.46 * (3.63, 5.29)		3.59 * (2.81, 4.37)	2.62 * (1.82, 3.41)		2.47 * (1.60, 3.34)	2.05 * (1.14, 2.97)		3.66 * (2.98, 4.34)	3.04 * (2.34, 3.75)
RT (days/week)			1.58 * (0.62, 2.54)			3.36 * (2.43, 4.28)			1.45 * (0.39, 2.51)			2.13 * (1.31, 2.95)
*R^2^*	11.5	29.7	31.0	9.1	20.3	26.8	6.1	11.0	12.1	10.0	24.7	28.0

* *p* < 0.05. All models are adjusted for sociodemographic variables (age, sex, ethnicity, education, household income, dog ownership, access to motor a vehicle, and tobacco use). MVPA: Moderate-to-vigorous intensity physical activity (days per week achieving 30-minutes of MVPA). RT: Resistance training (days per week doing weights, resistance training). PANES: Physical Activity Neighborhood Environment Scale. PPI: Park Perceptions Index. R^2^: Proportion of variance explained by the model. Walk Score^®^, PANES, and PPI examined separately from each other.
